# Investigating Random Linear Coding from a Pricing Perspective

**DOI:** 10.3390/e20080548

**Published:** 2018-07-25

**Authors:** Hailing Zhu, Khmaies Ouahada

**Affiliations:** Department of Electrical and Electronic Engineering Science, University of Johannesburg, Johannesburg 2006, South Africa

**Keywords:** bulk service, pricing, queuing theory, random network coding

## Abstract

In this paper, we study the implications of using a form of network coding known as Random Linear Coding (RLC) for unicast communications from an economic perspective by investigating a simple scenario, in which several network nodes, the users, download files from the Internet via another network node, the sender, and the receivers as users pay a certain price to the sender for this service. The mean packet delay for a transmission scheme with RLC is analyzed and applied into an optimal pricing model to characterize the optimal admission rate, price and revenue. The simulation results show that RLC achieves better performance in terms of both mean packet delay and revenue compared to the basic retransmission scheme.

## 1. Introduction

In the concept of network coding [1], encoding operations are performed on a group of data packets (e.g., by using the exclusive-OR (XOR) function) before they are forwarded towards their destinations, which departs from the conventional store-and-forward transmission paradigm. It is established in [1] (for linear network encoding) and [2] (for random linear network coding) that network coding benefits the throughput for multicast transmission by increasing the amount of data delivered per unit time. Since it is capable of increasing network throughput, improving robustness to channel errors and simplifying the design of distributed architectures, various applications based on the concept of network coding have been developed to improve throughput, reliability and energy efficiency in a number of related fields, such as multimedia applications [3,4]. In particular, Random Linear Coding (RLC) provides a fully distributed methodology for performing network coding [5]. With RLC, each node in the network independently selects random code vectors or coefficients to encode the data packets it receives into random linear combinations of the original data packets. These linear combinations are then transmitted with the associated code vectors until the receivers decode the original information packets using Gaussian elimination. By exploiting the broadcasting nature of the wireless medium, RLC has also been combined with opportunistic routing in wireless mesh networks to avoid node coordination, which is required by traditional opportunistic routing, while providing significant performance gains [6,7,8,9,10]. The common idea behind these applications is that a source node encodes information packets using RLC, all intermediate nodes also need to perform RLC to encode the random linear combinations that they hear from the source node or other intermediate nodes and send out these new random linear combinations until the receiver decodes the original information packets.

In this paper, we study the performance of RLC from an economic perspective by investigating a simple scenario where a network node, the receiver, downloads a file from another network node, the sender, and the receiver as a user pays a certain price to the sender for this service. Note that, in wireless mesh networks, this sender could be either a source node or an intermediate node. We compare two communication schemes: RLC, in which a group of packets is encoded and the random linear combinations are transmitted until the receiver successfully decodes all the original information packets, and the basic retransmission scheme, in which a single packet is transmitted continuously until a successful delivery is confirmed.

Recently, a great deal of effort has been devoted to understanding communication network economics by both the engineering and economic research communities. From a profit-maximizing service provider’s perspective, its aim is to maximize its revenue while inducing desirable user response by using price as a soft admission control mechanism. From a delay-sensitive user’s perspective, service delay is one of the main factors that affect his/her satisfaction with the service, which is normally expressed by net utility, besides price. In this paper, our analysis is based on a provider’s revenue optimization model with users’ compensated utility, which is a function of mean packet delay and price, integrated as a constraint.

Given the fact that with RLC all the original information packets have to be decoded together only after a destination node receives a sufficient number of encoded packets, the transmission delay (defined as the time between the moment when the packet arrives into a sender and the moment when the packet has been successfully delivered and decoded by a receiver) obviously differs from that caused in the conventional retransmission scheme. Shrader et al. in [11] analyze the queueing delay performance for two different block-based random RLC schemes: coding over a fixed blocksize and a variable blocksize, which adapts to the traffic load, for multicast transmission over packet erasure channels by modeling random coding of packets as a bulk-service queueing system, where packets are served and depart the queue in groups. It is shown that RLC can offer benefits over uncoded transmissions in terms of both throughput and delay if the blocksize is adapted to the traffic load. It is also suggested in [11] that bulk-service queueing , which has been applied in other works (e.g., [12,13,14]), for RLC performance analysis, is an appropriate model to capture the fact that randomly arriving packets are encoded, transmitted, and removed from the queue in groups corresponding to blocks.

Due to the impact of RLC on the delay performance, it is interesting to study the implications of RLC from an economic perspective. The objective of this paper is to study the mean packet delay with RLC and quantify the impact of RLC from a pricing perspective by comparing the optimal admission rate, the optimal price, and the corresponding expected delay as well as optimal revenue obtained by a node using RLC with those obtained from the basic retransmission scheme. In this paper, the $Geo/G^{K}/1$ bulk service queuing model presented by Shrader et al. is slightly modified. As a result, the processing of the bulk service can be compared with a simple and well-documented Geo/Geo/1 queuing model and an approximate but analytically tractable expression for the mean packet delay with RLC can be derived. Based on this approximate expression, we analyze the optimal admission rate, the optimal price and the corresponding expected delay when the sender node chooses to use RLC and compare them with those obtained from the basic retransmission scheme to study the performance of RLC from an economic point of view.

The rest of the paper is organized as follows: We briefly discuss the related works in Section 2. Section 3 describes the system model and introduces some basic notations. Section 4 focuses on the analysis of the mean packet delay with RLC adopting the method proposed in [13]. In Section 5, the revenue optimization problem is set up to characterize the optimal admission rate, the optimal price and the corresponding optimal delay. Numerical examples are given and comparisons between RLC and the basic retransmission are provided in Section 6. Section 7 contains concluding remarks and ideas for future work.

## 2. Related Work

A great deal of work has been done to gain a better understanding of the delay behavior of RLC, which is instrumental towards its successful application, especially in real-time applications. Lucani et al. in [15] investigated the mean time to complete the transmission of a block of packets to all receivers for broadcasting in time division duplexing channels with the use of random linear network coding by modeling the process as a Markov chain. It is shown that there is an optimal number of coded data packets to be transmitted back-to-back to minimize the mean time to complete transmission of the block of packets. Using the techniques discussed in [15], Lucani et al. further studied the performance of a systematic network coding approach over time division duplexing channels in terms of mean completion time taking the field size into consideration [16]. It is shown that the proposed systematic network coding scheme can provide the same or close to the same performance in terms of completion time as a random linear network coding scheme using a large field size. Cogill et al. [17] analyzed the delay of a simple RLC scheme for transmitting packets across a relay network, which is modeled as a continuous-time Markov chain, and provide an upper bound on the expected transmission time for RLC.

Eryilmaz et al. [18] studied a broadcast model with RLC involved and show that network coding offers better performance in terms of delay under the assumption that there is a fixed amount of data awaiting transmission at the sender node. However, in a realistic network setting, the number of packets awaiting transmission varies due to queuing effects when the channel is ready for transmission. Moreover, Shrader et al. [11] showed that random linear coding can offer benefits over uncoded transmissions in terms of both throughput and delay if the blocksize is adapted to the traffic load. Shrader et al. [12] developed a bulk-service queuing model for RLC, in which packets randomly arrive at a source node for transmission to a single destination and the number of packets used in encoding is a variable with a predefined maximum value. Based on this $Geo/G^{K}/1$ queuing model, an expression is derived for the steady-state probability distribution for the number of packets in the queue, by which the mean packet delay of RLC for such a unicast model can be computed approximately. Ravanbakhsh et al. [13] further extended the work of Shrader et al. [12] and computed the mean packet delay of RLC with an improved expression. Due to the complexity of the $Geo/G^{K}/1$ queuing model, there is no general expression for the mean packet delay derived in [12,13].

The economic aspects of data transmission systems with RLC involved are rarely studied. Our work is in line with the work of Ahmed et al. [19], who analyzed the economic gains obtained from RLC for a multicast set introduced in [18]. In [19], a single base station broadcasts incoming files to multiple receivers and the base station sets a price per receiver and per file to maximize its profit. By studying the mean file completion times of network coding and scheduling strategies, Ahmed et al. suggested that network coding offers significant economic gains as opposed to scheduling. Our work differs from [19] by considering a unicast model, which is more realistic than a broadcast model. Moreover, we focus on the queuing delay instead of assuming that there is a fixed amount of data awaiting transmission.

## 3. System Model

Assume there is a group of users, where each downloads a certain file consisting of a certain amount of packets from a sender. In this file transfer process, information packets demanded by a user arrive at the sender’s queueing system and the sender transmits these information packets to the user accordingly. This file transfer process can be viewed as a “one-sender one-unicast” scenario. As suggested in [12,13], this transmission can be modeled as a queuing system where packets demanded by the user arrive to the sender according to a Bernoulli process with a rate λ, meaning the number of new packets arrive is either 1 or 0 with probabilities of λ or 1−λ, respectively, during a given time slot. For the sake of simplification, λ is referred to as the arrival rate for the rest of the paper. Upon arrival at the sender, the packet is stored in the buffer (queue) of the sender to await encoding and transmission. Assume that the sender has unlimited buffer memory. In each time slot, a packet is transmitted from the sender to a user with reception probability μ, 0<μ≤1. Thus, the average service time for a single packet is $\frac{1}{\mu }$ time slots.

Assume further that there is no delay between sending a packet and receiving an acknowledgment if a packet has been successfully received, meaning that only the queuing delay is being considered in this paper. In other words, the mean packet delay is defined as the average time between the moment when the packet arrives in the queue and the moment when the packet has been successfully delivered and decoded.

**Figure 1:**
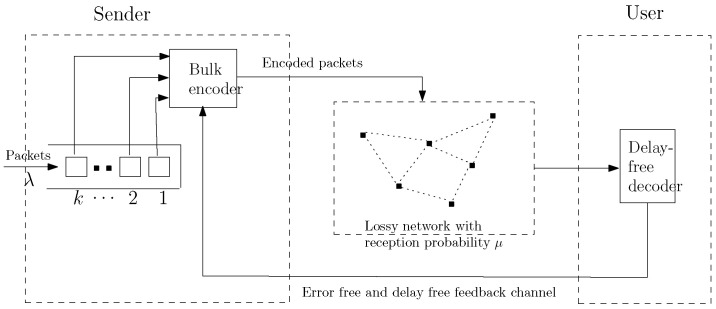
System model [13].

With *RLC*, the encoding and transmission are performed on groups of packets as shown in Figure 1. In reality, wireless channels are affected by impairments, such as multipath and shadow fading. In this paper, we assume the channel sending packets from the sender to the user as a packet-erasure channel where a packet transmitted by the sender is successfully delivered to the user with probability μ. As suggested in [12,13], this data transmission system can be represented by a discrete-time bulk-service queuing model. In [12,13], the number of packets used in encoding and transmission is a variable and must be smaller or equal to a maximum value *K*. This bulk service is modeled as a discrete-time $Geo/G^{K}/1$ queue. In this paper, we present a slightly different queuing model, where there is no maximum value limit. In other words, once the channel becomes free, all the packets $I_{1},\ldots ,I_{k}$ waiting in the queue, instead of only k≤K packets, are selected for encoding and transmission.

The set $\left\{I_{1},\ldots ,I_{k}\right\}$ is referred to as a bulk. Note that *k* is a variable. The sender generates encoded packets $I_{j}^{\prime }$ based on the bulk. These *k* uncoded packets are called message packets or native packets and each encoded packet $I_{j}^{\prime }$ is a random linear combination of these message packets. Denote an encoded packet by

$$I_{j}^{\prime }=\sum_{i=1}^{k}c_{ji}I_{i},$$

where $c_{ji}$ are random coefficients picked from some field F, and $\bar{c_{j}}=\left(c_{j1},c_{j2},\ldots ,c_{jk}\right)$ is called the code vector of the encoded packet $I_{j}^{\prime }$. The code vector describes how to generate the encoded packet from the message packets. It needs to be sent along with the encoded packet as side information in its header. If the message packets are sufficiently large, the overhead to contain the code vectors is negligible.

The sender proceeds to generate and send randomly encoded packets until the receiver node has successfully decoded all of the message packets. It has been proven in [20] that, when coding is performed in a very large field F, for a group of *k* message packets, the probability that a receiver can decode the *k* message packets upon successful reception of *k* encoded packets is close to 1. With this condition, it makes sense to compare the bulk service described above with a Geo/Geo/1 queuing system, since for both queuing systems transmission of *k* message packets will be completed when the channel has successfully delivered *k* packets.

To facilitate analysis, as in [14,18,21,22], we assume that RLC is performed over a sufficiently large field size, so that the expected number of successfully received packets at the receiver, to decode the original *k* data packets, is *k*. Thus, when the receiver node has successfully collected *k* linearly independent encoded packets $\left\{I_{1}^{\prime },\ldots ,I_{k}^{\prime }\right\}$, all of the message packets $\left\{I_{1},\ldots ,I_{k}\right\}$ can be decoded using simple matrix inversion:

$$\left(\begin{array}{c} I_{1}\\ \vdots \\ I_{k} \end{array}\right)={\left(\begin{array}{ccc} c_{11} & \ldots & c_{1k}\\ \vdots & \ddots & \\ c_{k1} & \ldots & c_{kk} \end{array}\right)}^{-1}\left(\begin{array}{c} I_{1}^{\prime }\\ \vdots \\ I_{k}^{\prime } \end{array}\right),$$

where $I_{i}$ is a message packet, and $I_{i}^{\prime }$ is a linearly independent encoded packet whose code vector is $\bar{c_{i}}=\left(c_{i1},c_{i2},\ldots ,c_{ik}\right)$. As soon as the receiver node decodes the group of message packets, it sends an acknowledgment to the sender to allow it to move to the next group of packets. After receiving the acknowledgment confirming the successful delivery, the sender processes the next group of packets. Note that, in the unicast scenario considered in this paper, transmission of a group of encoded packets is completed as soon as the user successfully decodes the group of encoded packet. Conversely, in a broadcasting model where a sender broadcasts the encoded packets to a set of receivers, transmission of a group of encoded packets completes until all receivers successfully decode the group of encoded packets.

## 4. User Utility

The encoding and transmission are viewed as a service, for which a user needs to pay a certain price. According to [23], the user will decide to use the service as long as its net utility (utility minus waiting cost) minus the price charged by the sender, which is called compensated utility, is positive. Denote the utility that the user will derive from receiving a packet from the sender by *U*, and the sender charges a price *p* per packet. Then, the corresponding compensated utility, U, is given by:

(1) U=U−p−βD

where *D* is the expected delay experienced by each packet, and β is a constant converting delay from time units into money units. The parameter β reflects the delay sensitivity of the user or the user’s application: the higher the value of β, the more sensitive the user’s application to delay. For example, multimedia services with stringent delay requirements will have high values of β. Thus, βD can be viewed as a waiting cost of a service that spends a delay *D* and the parameter β can be interpreted as a waiting cost per unit time.

A user will use the service if and only if U>0. Thus, the effective arrival rate λ is given by

(2) $$\begin{array}{cc} \lambda & =\Lambda P(U>0)\\ & =\Lambda P(U-p-\beta D>0)\\ & =\Lambda \int_{U>p-\beta D}f\left(u\right)du, \end{array}$$

where Λ is a potential arrival rate without considering the price charged by the sender and f(u) is the probability density function of *U*. Users are heterogeneous in terms of their valuations on the service. We assume that user valuations are upper bounded by α. To reflect the range of valuations in the population of users in a simple manner to allow for analytical tractability, as suggested in [24,25], each user is characterized by a valuation that is an independent random variable uniformly distributed over the interval [0,α]. This assumption corresponds to the commonly used assumption in the literature [26,27,28] where users are characterized by user types that are uniformly distributed on the interval [0,1]. Thus, Equation (2) can be simplified to

(3) $$\frac{1}{\alpha }\left(\alpha -p-\beta D\right)=\frac{\lambda }{\Lambda }.$$

With the condition that, when *k* message packets are being encoded over a sufficiently large field with RLC and transmitted, the receiver node can recover all of the *k* message packets after successfully receiving *k* encoded packets, Ravanbakhsh et al. [13] proposed a method to analyze the delay *D* for each packet by comparing the processing of the bulk service directly with a Geo/Geo/1 queuing system. The Geo/Geo/1 queuing model, which has been analyzed in [29], is used to model the basic retransmission scheme in [12,13]. For the basic retransmission scheme, the average waiting time $W_{RE}$ and the mean packet delay $D_{RE}$ are given by

(4) $$\left\{\begin{array}{c} W_{RE}=\frac{\lambda (1-\mu )}{\mu (\mu -\lambda )},\\ D_{RE}=\frac{1}{\mu }+W_{RE}=\frac{1-\lambda }{\mu -\lambda }. \end{array}\right.$$

Note that Equation (4) holds provided that μ>λ.

According to the analysis in [13], for a bulk of encoded packets $I_{i}^{\prime },I_{2}^{\prime },\cdots ,I_{k}^{\prime }$, a fictitious waiting time for each packet in the bulk is equal to the waiting time that a packet would experience in a Geo/Geo/1 queuing system, and the modified service time for each packet $I_{j}^{\prime }$ is (k−j+1)/μ. Thus, for *k* message packets, the mean packet delay is given by [13]

$$D_{I_{j}^{\prime }}=W_{RE}+\frac{1}{k}\frac{\sum_{i=1}^{k}i}{\mu }=\frac{\lambda (1-\mu )}{\mu (\mu -\lambda )}+\frac{k+1}{2\mu }.$$

In this paper, we use the same method to analyze the queuing system model described in Section 3. Then, with RLC, the expected delay experienced by each packet is given by

(5) $$D_{RLC}=\frac{\lambda (1-\mu )}{\mu (\mu -\lambda )}+\frac{\sum_{k=1}^{\infty }B_{k}\left(k+1\right)}{2\mu },$$

where $B_{k}$ is the probability that the bulk is of length *k*.

Here, Equation (5) can be written as

(6) $$\begin{array}{cc} D_{RLC} & =\frac{\lambda (1-\mu )}{\mu (\mu -\lambda )}+\frac{\sum_{k=1}^{\infty }B_{k}k+\sum_{k=1}^{\infty }B_{k}}{2\mu }\\ & =\frac{\lambda (1-\mu )}{\mu (\mu -\lambda )}+\frac{\sum_{k=1}^{\infty }B_{k}k+1}{2\mu }, \end{array}$$

where $\sum_{k=1}^{\infty }B_{k}k$ is the expected number of packets in the queue immediately after the previous bulk has been successfully delivered. Denote $\sum_{k=1}^{\infty }B_{k}k$ by $\bar{S}$. Note that $\bar{S}$ may not be the same as the average number in the system at an arbitrary point in time. To facilitate analysis, we use $\bar{S}$ as an approximation to the average number in the system at an arbitrary point in time. According to Little’s law,

$$\bar{S}=\lambda D.$$

Multiplying by λ on both sides of Equation (6) and substituting λD and $\sum_{k=1}^{\infty }B_{k}k$ with $\bar{S}$, we obtain

(7) $$\bar{S}=\lambda \left[\frac{\lambda (1-\mu )}{\mu (\mu -\lambda )}+\frac{\bar{S}+1}{2\mu }\right].$$

Thus,

$$\bar{S}=\frac{\lambda (\lambda -2\lambda \mu +\mu )}{\left(\mu -\lambda )(2\mu -\lambda \right)},$$

which implies that

(8) $$D_{RLC}=\frac{\lambda -2\lambda \mu +\mu }{\left(\mu -\lambda )(2\mu -\lambda \right)}.$$

Note that Equation (8) holds provided that μ>λ.

## 5. Revenue Optimization

The revenue generated by the sender per unit time is given by Π=pλ. We evaluate the economic impact of RLC by comparing it with the basic retransmission scheme. Thus, the sender’s revenue maximization problem can be written as

(9a)Maximize:pλ(9b)Subjectto:1α(α−p−βD)=λΛ;(9c)λ<μ,

where

$$\begin{array}{cc} D_{RLC}=\frac{\lambda -2\lambda \mu +\mu }{\left(\mu -\lambda )(2\mu -\lambda \right)}, & \mathrm{for}\;\mathrm{RLC};\\ D_{RE}=\frac{1-\lambda }{\mu -\lambda }, & \mathrm{for}\;\mathrm{basic}\;\mathrm{retransmission}. \end{array}$$

Rearranging Equation (9b) yields

(10) $$p=\alpha -\beta D-\frac{\alpha \lambda }{\Lambda }.$$

Substituting Equation (10) into Equation (9a), we rewrite the sender’s revenue as

(11) $$\Pi =\alpha \lambda -\beta \lambda D-\frac{\alpha \lambda^{2}}{\Lambda }.$$

Differentiating the revenue function in Equation (11) with respect to λ, we obtain

(12) $$\frac{\partial \Pi }{\partial \lambda }=\alpha -\beta D-\beta \lambda D^{\prime }-\frac{2\alpha }{\Lambda }\lambda .$$

where

$$\begin{array}{c} D_{RLC}^{\prime }=\frac{5\mu^{2}-4\mu^{3}-\lambda^{2}+2\mu \lambda^{2}-2\mu \lambda }{{\left(\mu -\lambda \right)}^{2}{\left(2\mu -\lambda \right)}^{2}},\;\mathrm{for}\;\mathrm{RLC};\\ D_{RE}^{\prime }=\frac{1-\mu }{{\left(\mu -\lambda \right)}^{2}},\;\mathrm{for}\;\mathrm{basic}\;\mathrm{retransmission}. \end{array}$$

Equating $\frac{\partial \Pi }{\partial \lambda }$ to 0, expressions for the optimal arrival rates for RLC and basic retransmission, $\lambda_{opt_{RLC}}$ and $\lambda_{opt_{RE}}$, are given, respectively, by

(13) $$1-\frac{2}{\Lambda }\lambda_{opt_{RLC}}-\frac{\beta }{\alpha }D_{opt_{RLC}}-\frac{\beta }{\alpha }\lambda_{opt_{RLC}}D_{opt_{RLC}}^{\prime }=0,$$

where

$$\begin{array}{c} D_{opt_{RLC}}=\frac{\lambda_{opt_{RLC}}-2\lambda_{opt_{RLC}}\mu +\mu }{\left(\mu -\lambda_{opt_{RLC}}\right)\left(2\mu -\lambda_{opt_{RLC}}\right)};\\ D_{opt_{RLC}}^{\prime }=\frac{5\mu^{2}-4\mu^{3}-\lambda_{opt_{RLC}}^{2}+2\mu \lambda_{opt_{RLC}}^{2}-2\mu \lambda_{opt_{RLC}}}{{\left(\mu -\lambda_{opt_{RLC}}\right)}^{2}{\left(2\mu -\lambda_{opt_{RLC}}\right)}^{2}}, \end{array}$$

and

(14) $$1-\frac{2}{\Lambda }\lambda_{opt_{RE}}-\frac{\beta }{\alpha }D_{opt_{RE}}-\frac{\beta }{\alpha }\lambda_{opt_{RE}}D_{opt_{RE}}^{\prime }=0,$$

where

$$\begin{array}{c} D_{opt_{RE}}=\frac{1-\lambda_{opt_{RE}}}{\mu -\lambda_{opt_{RE}}};\\ D_{opt_{RE}}^{\prime }=\frac{1-\mu }{{\left(\mu -\lambda_{opt_{RE}}\right)}^{2}}. \end{array}$$

Note that Equations (13) and (14) hold provided that $\mu >\lambda_{opt_{RE}}$ and $\mu >\lambda_{opt_{RE}}$, respectively. The solutions to Equations (13) and (14), $\lambda_{opt_{RLC}}$ and $\lambda_{opt_{RE}}$, can be used as the optimal admission rates for the sender when it chooses to use RLC or the basic transmission scheme, respectively.

Subsequently, according to Equation (9b), the corresponding optimal prices $p_{opt_{RLC}}$ and $p_{opt_{RE}}$ are given, respectively, by:

(15) $$p_{opt_{RLC}}=\alpha -\beta D_{opt_{RLC}}-\frac{\alpha \lambda_{opt_{RLC}}}{\Lambda },$$

and

(16) $$p_{opt_{RE}}=\alpha -\beta D_{opt_{RE}}-\frac{\alpha \lambda_{opt_{RE}}}{\Lambda }.$$

For both cases, with the optimal admission rates and the corresponding optimal prices, the resulting sender’s revenues, which are given, respectively, by

(17) $$\pi_{opt_{RLC}}=p_{opt_{RLC}}\lambda_{opt_{RLC}}$$

and

(18) $$\pi_{opt_{RE}}=p_{opt_{RE}}\lambda_{opt_{RE}}$$

are maximized.

However, Equations (13) and (14) are difficult to solve mathematically. We study the impact of RLC in terms of the optimal admission rate $\lambda_{opt}$, the optimal price $p_{opt_{RLC}}$, the expected delay $D_{RLC}$ and the optimal revenue $\Pi_{opt}$, through the following numerical examples.

## 6. Numerical Examples

**Figure 2:**
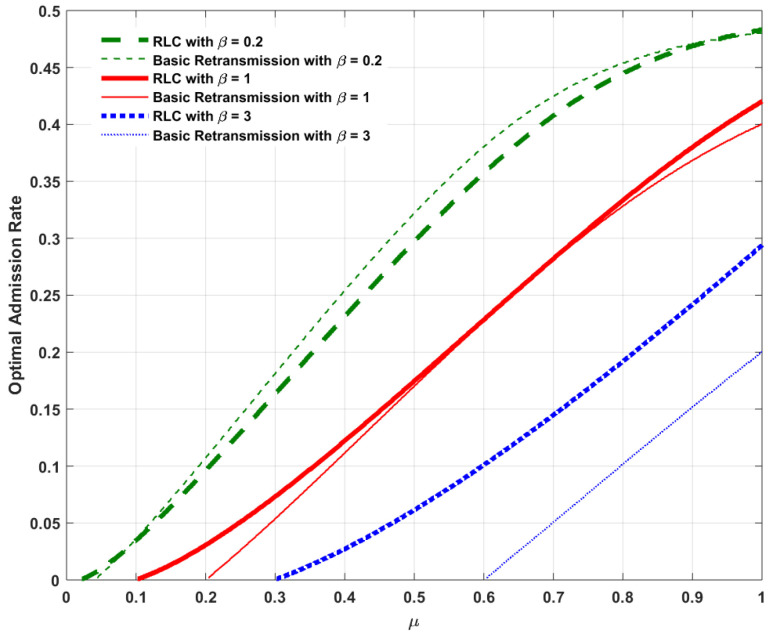
Comparison of optimal admission rate $\lambda_{opt}$ between Random Linear Coding (RLC) and basic retransmission against reception probability μ.

**Figure 3:**
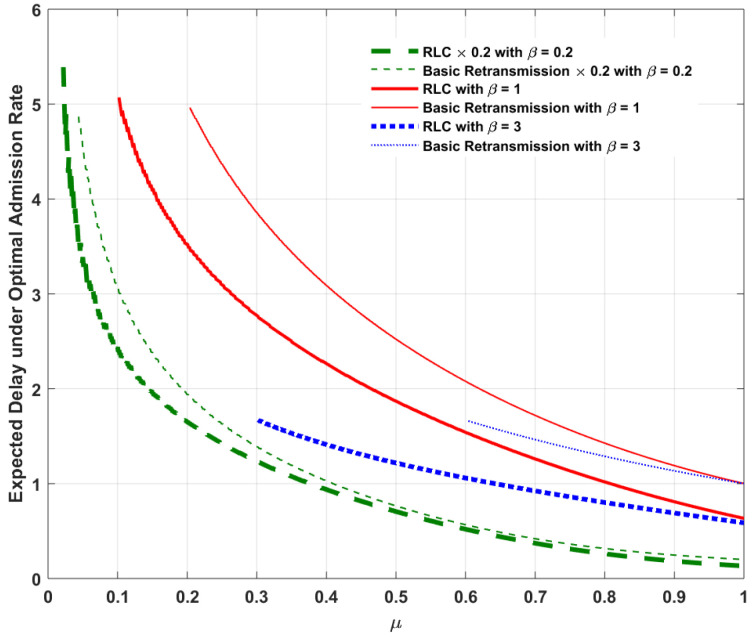
Comparison of expected delay under the optimal admission rate $\lambda_{opt}$ between RLC and basic retransmission against reception probability μ. Note: The expected delays for β=0.2 are scaled by a factor 0.2 to display all curves clearly in the figure.

**Figure 4:**
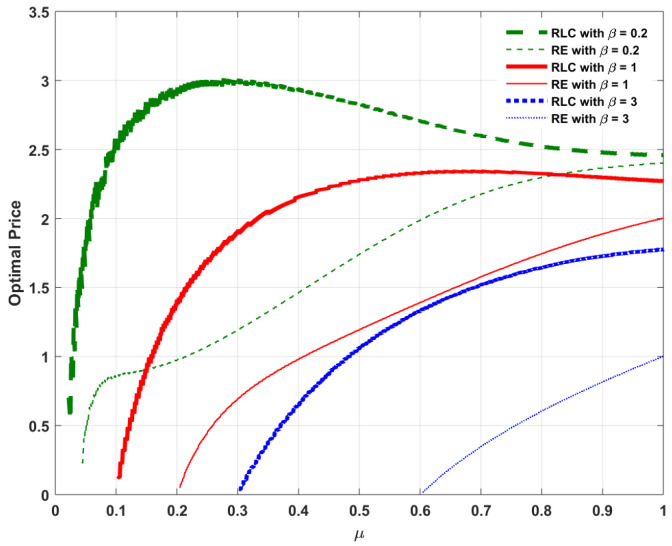
Comparison of optimal price $p_{opt}$ between RLC and basic retransmission against reception probability μ.

**Figure 5:**
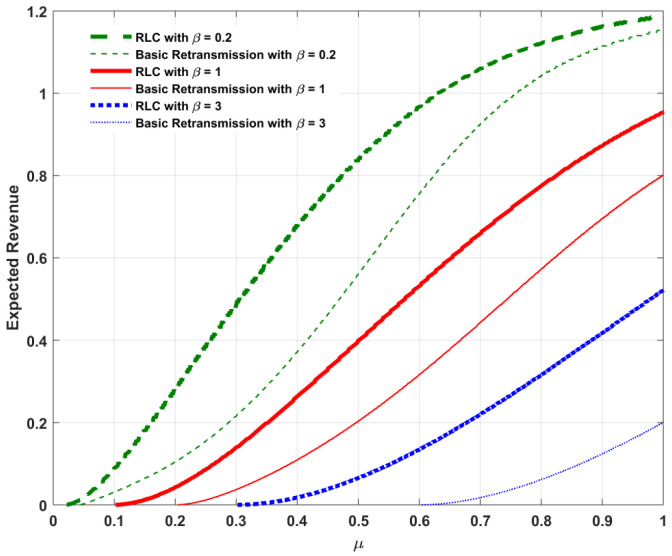
Comparison of optimal revenue $\Pi_{opt}$ between RLC and basic retransmission against reception probability μ.

Assume Λ=1 and α=5 as listed in Table 1. With the assumption Λ=1, we focus our attention on a high-traffic load service model to investigate the behavior of the admission rate under the impact of RLC. For ease of illustration, Figure 2, Figure 3, Figure 4 and Figure 5 plot the optimal admission rate, the corresponding mean packet delay under the optimal admission rate, the optimal price and the corresponding optimal revenue for RLC and the basic retransmission scheme as a function of μ with β=0.2, β=1 and β=3, respectively.

The following can be observed:

- In Figure 2, for both RLC and the the basic retransmission scheme, the optimal admission rate is proportional to the reception probability. The reason is that, the lower the reception probability, the longer the service time per packet, which results in a lower admission rate needed to avoid potential congestion. In addition, considering the pricing method involved as an admission control mechanism, both schemes allow lower admission rates when the user is more delay-sensitive. In addition, the optimal admission with the basic retransmission scheme is slightly higher when the user is more tolerant to delay (e.g., β=0.2), whereas RLC allows a higher optimal admission rate when the user is more delay-sensitive (e.g., β=3).
- In Figure 3, the corresponding mean packet delay with RLC is lower than that of the basic retransmission scheme, under their respective optimal admission rates. This can be explained as follows: when comparing $D_{RLC}$ with $D_{RE}$, it can be found that, if ${\left(1-\lambda \right)}^{2}\leq 1-\mu$, we have $D_{RLC}\leq D_{RE}$. For an arbitrary reception probability in the feasible range, the optimal admission rate with RLC, $\lambda_{opt_{RLC}}$, and that for the basic retransmission scheme, $\lambda_{opt_{RE}}$, clearly satisfy the condition ${\left(1-\lambda \right)}^{2}\leq 1-\mu$. It can also be seen that, both RLC and the basic retransmission scheme enable lower expected mean packet delays when the user is more delay-sensitive due to the effect of optimal pricing on the delay-sensitive user. This result can be explained as follows: when the user is more delay sensitive, i.e., the waiting cost per unit time β is higher, which in turn could lead to a higher waiting cost βD, accordingly, a lower delay *D* is resulted from a lower admission rate (as shown in Figure 2) induced by a higher price (as shown in Figure 4) to balance the waiting cost in such a way that the compensated utility U>0 is guaranteed according to Equation (3).
- In Figure 4, for both cases, the optimal price is an increasing function of the reception probability. It is intuitive that the queuing delay is decreasing with the increase of the reception probability, as illustrated in Figure 3. According to Equation (1), as the queuing delay decreases, the sender can increase the price while guaranteeing the compensated utility U>0. In addition, since $D_{RLC}\leq D_{RE}$, under the respective optimal admission rates of both cases, $p_{RLC}$ can be higher than $p_{RE}$ while guaranteeing the compensated utility U>0. Furthermore, for both cases, the optimal price reduces as a result of increased waiting cost βD when the user is more delay-sensitive.
- In Figure 5, for both cases, the optimal revenue, which is the product of the corresponding optimal admission rate and optimal price, increases as the reception probability increases in a quasi-linear fashion. As expected, the optimal revenue with RLC is higher than that for the basic retransmission scheme under their respective optimal admission rates.

## 7. Conclusions

We have investigated a transmission scheme with RLC involved from a pricing viewpoint in a unicast setting, in which the message packets demanded arriving at the sender according to a random arrival pattern, are encoded and then transmitted to a single destination, taking user’s delay-sensitivity into consideration. It is shown that the optimal admission rate, the optimal price and the corresponding optimal revenue depend on the quality of the transmission channel. The optimal revenue is a monotonically increasing function of the reception probability. In comparison with the basic retransmission scheme, it is observed that RLC offers better performance in terms of both mean packet delay and revenue. In addition, compared to the basic retransmission scheme, RLC allows a higher admission rate when the user is more delay-sensitive.

In this paper, to facilitate the analysis, we assume a sufficiently large field size so that any *k* vectors of coding coefficients are linearly independent with a high probability, i.e., the expected number of successfully received packets to decode the original *k* packets is *k*. While the assumption not only significantly simplifies the analysis but also leads to better delay performance, the field size is limited in practice due to the encoding and decoding complexity. However, smaller finite field sizes result in a non-negligible probability that the vectors of coding coefficients collected by a user are linearly dependent, even if their number is larger than *k*, leading to a lower probability of successful decoding. As a result, with a finite field size, decoding probability should be derived and integrated into the delay analysis. This would be an extension for future work.

Note that the unicast setting discussed in this paper is a general model in which we only assume a lossy channel with certain reception probability. Exploiting the properties of networks (i.e., wireless mesh networks) and taking advantage of them when applying RLC may result in an improved delay performance. Therefore, to analyze a more complicated and realistic system model, for instance, an opportunistic wireless network with RLC, would be another interesting extension. 

## Figures and Tables

**Table 1:** Simulation setting.

Parameter	Description	Value
Λ	Potential Arrival Rate	1
α	Maximum User Utility	5
β	Waiting Cost Per Unit Time	0.2, 1, 3
